# Chagas disease in Canadian blood donors: 15 years of selective testing

**DOI:** 10.1111/vox.70299

**Published:** 2026-06-01

**Authors:** Sheila F. O'Brien, Mindy Goldman, Antoine Lewin, Samra Uzicanin, Wenli Fan, Steven J. Drews

**Affiliations:** ^1^ Epidemiology & Surveillance Canadian Blood Services Ottawa Ontario Canada; ^2^ School of Epidemiology & Public Health University of Ottawa Ottawa Ontario Canada; ^3^ Donation and Policy Studies Canadian Blood Services Ottawa Ontario Canada; ^4^ Department of Pathology & Laboratory Medicine University of Ottawa Ottawa Ontario Canada; ^5^ Medical Affairs and Innovation, Héma‐Québec Montréal Québec Canada; ^6^ Department of Mathematics, Faculty of Science University of Sherbrooke Sherbrooke Québec Canada; ^7^ Microbiology Canadian Blood Services Edmonton Alberta Canada; ^8^ Department of Laboratory Medicine & Pathology, Faculty of Medicine & Dentistry University of Alberta Edmonton Alberta Canada

**Keywords:** blood donation, blood donor, Chagas disease, policy, *Trypanosoma cruzi*

## Abstract

**Background and Objectives:**

Chagas disease (*Trypanosoma cruzi*), prevalent in Mexico, Central and South America, can be transfusion‐transmitted. Selective serological testing of blood donors was implemented over 15 years ago. We describe the trends in infections and characteristics of donors selected for testing.

**Materials and Methods:**

Donor selection for testing was based on birth in Latin America and/or mother/grandmother born in Latin America and/or travel to Latin America (30 days duration at Héma‐Québec [HQ], 6 months at Canadian Blood Services [CBS]). All first‐time donors (2010–2024) were analysed. Frequencies were compiled by gender, age group, region and residential material deprivation and ethnocultural composition indices. Multiple logistic regression compared donors with risk factors (tested) versus those without risk factors (not tested).

**Results:**

Of the 67,490 tested donors, most were born in Mexico, Central or South America (71% at CBS). Of the 27 donors positive for *T. cruzi* antibodies, more were from Ontario, materially deprived and ethnocultural concentrated neighbourhoods. One had mother/grandmother birth, but none had travel as the sole risk factor. Tested donors were similar to non‐tested donors except more likely to be aged 30–49 (odds ratio [OR] 1.4, 1.37–1.44 CBS, 1.57, 1.51–1.63 HQ) and live in more ethnoculturally concentrated neighbourhoods (OR 2.09, 2.01–2.18 CBS, 3.46, 3.29–3.64 HQ).

**Conclusion:**

Birth in Latin America captures most *T. cruzi* antibody positive donations. Mother/grandmother risk occasionally yields a positive donation, but travel history does not. Infections in donors are rare, but selective testing interdicts a small risk of *T. cruzi* positive blood products being released into inventory.

**Highlights**

- In Canada, selective testing of blood donors based on risk factors for Chagas disease (*Trypanosoma cruzi* infection) has been in place for over 15 years.
- Serological evidence of Chagas disease infection in Canadian blood donors is rare. Most donors with positive tests were born in Mexico, Central or South America, although there was one donor whose only risk factor was mother/grandmother birth. No donors with infections had sole travel risk.
- Selective testing for Chagas disease prevents a small risk to the blood supply.

## INTRODUCTION

Chagas disease is a zoonotic infection of the parasite *Trypanosoma cruzi*, endemic in Mexico, Central and South America. Infection in humans usually occurs from the bite of a triatomine insect which releases infected faeces or urine as it feeds, infecting the host through the bite wound or mucous membranes. Vector to human transmission is more likely when triatomines infest substandard housing (such as with dirt floors, adobe walls and thatched roofs) and food‐borne contamination in rural parts of Latin America. Due to within‐country migration of people from rural to urban areas, Chagas disease has become more common in urban areas in endemic countries. Chagas disease can also be transmitted from mother to child during birth, and by blood transfusion although both of these routes are less common [1, 2]. Chagas disease has a short acute, often symptomatic, phase with high parasitaemia followed by an asymptomatic indeterminate phase with low or undetectable parasitaemia, and decades later, 30%–40% may develop serious cardiac or gastrointestinal disease [3]. Chagas disease can be cured in the acute stage of infection, but treatment is less effective in later stages.

In non‐endemic parts of the world, Chagas disease is associated with immigrants from endemic areas. A handful of transfusion‐transmitted cases (associated with apheresis and whole blood‐derived platelets) have been documented in non‐endemic countries when blood donation testing was not in place including three in Canada [4, 5]. Most non‐endemic countries that have implemented testing of blood donations have opted for selective testing of donors with risk factors, such as the United Kingdom, France, Switzerland, Spain, Italy and Canada [6, 7]. In the United States, all donors are tested one time to address the small risk of sylvatic autochthonous infections in the southern parts of the United States, although most infections in the United States are among immigrants from Latin America [8]. Reduviidae (assassin bugs) have been identified within Canada, but members of the blood‐feeding Triatominae have not been recorded, and there are no sylvatic autochthonous cases [9, 10]. Selective testing of at‐risk donors was implemented in 2009 in Québec. All other regions initially did not make platelets from donors with risk factors as of 2009 before implementing selective testing in 2010. We have previously reported that in the first year of testing, 13 first‐time tested donors had antibody‐positive results, of whom 11 were born in South America and two were born in Canada but had mothers born in Latin America and had travel [11]. We updated our results to October 2012 with an additional two antibody‐positive donors [12].

Surveillance of Chagas disease in blood donors continues to be important to understand trends in infection positive donors and to evaluate the ongoing impact of the testing policy. Here we report the number of first‐time donors who were tested for Chagas disease over an additional 12 years (from 2010 to 2024) across all Canadian provinces and describe the demographic composition of those tested and those positive.

## MATERIALS AND METHODS

There are two blood operators that collect and distribute labile blood products in Canada. Canadian Blood Services (CBS) collects blood donations in all provinces except Quebec. Héma‐Québec (HQ) is responsible for blood collection in the province of Quebec.

### Selective testing algorithm

#### Canadian Blood Services

At CBS, selective testing for *T. cruzi* antibody is based on three questions:

1. Have you spent a total of 6 months or more in a continuous period in Mexico, Central America or South America?
2. Were you born in Mexico, Central America or South America?
3. Was your mother or grandmother born in Mexico, Central America or South America?

Throughout the study period, all first‐time donors were asked all three questions. Up until July 2016, all donors were asked these questions each time they donated. From July 2016 forward, frequent donors could complete an abbreviated questionnaire and were only asked about travel. Donors who answer yes to any of these questions are asked about the country in question to ascertain that it was Mexico or a country in Central or South America. A tick box format is used to document Mexico, Central or South America, but documentation of the specific country is incomplete. Donors whose only risk was their grandmother's birthplace were only tested if it was their maternal grandmother (not if only paternal). Prior to July 2016, donors were tested on each donation if they answered yes to any question. As of 2016, donors were tested only once if they were born in, or their mother or maternal grandmother was born in Mexico, Central America or South America, but were tested on each donation if they answered yes to the travel question.

#### Héma‐Québec

At HQ, donors are asked two questions:

1. Have you ever spent time in Latin America (including Mexico) for 30 consecutive days or more?
2. Were you or was your mother or maternal grandmother born in Latin America, including Mexico?

*Note*: Additional questions are asked if donors answer yes to either question to determine the country and are only tested if the country was in Mexico or in Central or South America.

Donors who answered yes to either question were tested, but additional data on country of birth were not collected. As of 2015, donors were tested only once if they were born in, or their mother or maternal grandmother was born in Mexico, Central America or South America but were tested on each donation if they answered yes to the travel question.

At both CBS and HQ, information about Chagas disease and testing was included in the pre‐donation reading materials. Donors are also asked if they have a history of Chagas disease and are permanently deferred if they have.

### Laboratory methods

At CBS testing was carried out using the Abbott PRISM Assay (Abbott Laboratories, Wiesbaden, Germany) until March 2020, then the Abbott Architect i2000SR (Abbott Laboratories) up until 1 April 2025, thereafter on Roche Elecsys (Roche Laboratories, Basel, Switzerland). Repeat reactive samples underwent supplemental/confirmatory testing at the National Reference Centre for Parasitology (McGill University Centre for Tropical Disease, Montreal, Quebec, Canada) using an immunoblot (IB) assay and PCR assay updated to real‐time PCR [13, 14, 15]. Donors were considered confirmed positive if any of the supplemental/confirmatory assays were positive.

At HQ, the implementation of Chagas disease screening was achieved through the progressive integration of screening and confirmatory methodologies over time. Prior to in‐house implementation, Chagas testing and confirmation for HQ were performed externally in the United States, beginning in March 2009 at the American Red Cross (ARC) using the Ortho enzyme linked immunosorbent assay (ELISA) assay (Ortho‐Clinical Diagnostics). In September 2011, ARC changed to the Abbott PRISM immunochemiluminescence assay (Abbott Laboratories) for primary screening, while confirmation continued to rely on Ortho ELISA. In 2014, HQ implemented on‐site Chagas disease screening, utilizing the Abbott PRISM immunochemiluminescence assay for primary screening. Confirmatory testing remained external and was performed at ARC National Testing Laboratories in Charlotte, North Carolina, using the Abbott excreted‐secreted antigens (ESA) enzyme strip assay (Abbott Laboratories). In October 2017, HQ transitioned to the Abbott PRISMnEXT platform (Abbott Laboratories) while maintaining the same confirmatory algorithm. Beginning in July 2017, confirmatory samples were analysed by Creative Testing Solutions (CTS) in Phoenix. On 5 December 2022, HQ replaced Abbott PRISMnEXT with the Roche Cobas pro e801 Elecsys Chagas immunochemiluminescence assay (Basel, Switzerland) while retaining CTS‐based ESA confirmatory testing.

### Donor data

All first‐time donations from July 2010 (January 2010 for HQ) to December 2024 were extracted from the donor databases of each blood operator, as well as age, gender, province, Chagas risk question answers and test results. Two indices based on residential dissemination area (derived from postal code) that categorized donors into quintiles of the general population: The Pampalon material deprivation index (MDI) and the CanMarg ethnocultural composition index [16, 17]. Material deprivation is associated with an insecure job situation, insufficient income and low education. Ethnocultural composition is associated with the proportion of people in the neighbourhood who are recent immigrants and/or visible minorities.

Research ethics review was waived as this was analysis of operational data.

### Statistical analysis

The numbers of donors who answered yes to Chagas risk questions were tallied by question combination and by demographic variables and percentages calculated. Logistic regression was performed with donors with risk factors (tested) versus those without risk factors (not tested) as the dependent variable and demographic variables as the independent variables. The donors who tested confirmed positive for Chagas disease were listed with demographic factors and risk question responses. All analyses were carried out using SAS version 9.4 (SAS Institute Inc., Cary, NC).

## RESULTS

**FIGURE 1:**
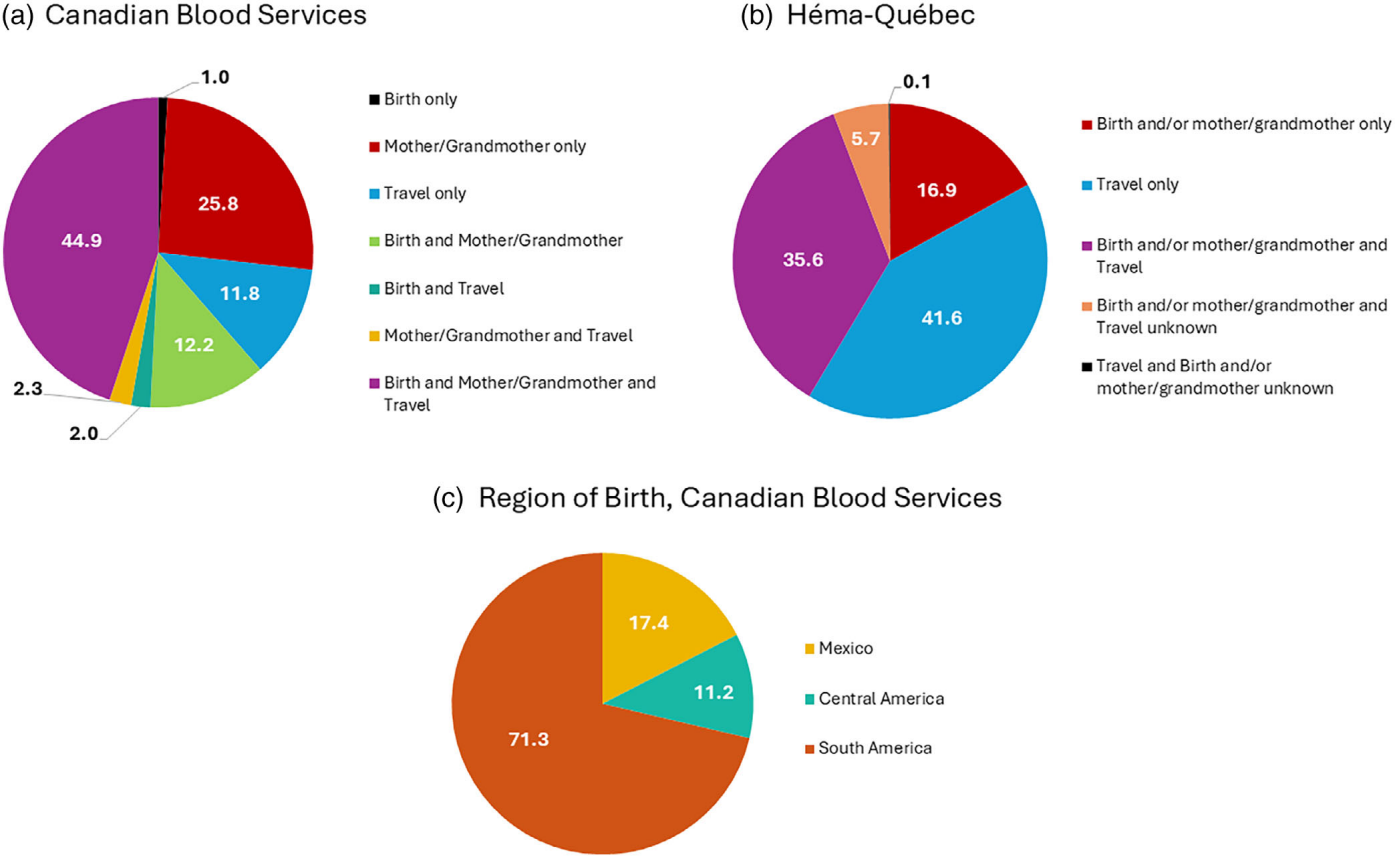
The percentages of first‐time allogeneic donors tested for *Trypanosoma cruzi* antibodies (a, b) by their risk factors, 2010–2024, Canadian Blood Services (CBS), 2015–2024, Héma‐Québec (HQ) and (c) the breakdown of first‐time allogeneic donors born in Latin America by region of birth, 2016–2024 (CBS only). For CBS, region of birth was not available in donor records prior to July 2016. For HQ, birth or mother/grandmother born in Latin America are asked as one question and are combined. The CBS travel questions was time ever spent in Latin America for six consecutive months; HQ 1 month. Allogeneic donors include whole blood, plasma for transfusion and platelet (99.93% are whole blood).

There were 67,460 (42,546 CBS and 21,914 HQ) first‐time donors tested for *T. cruzi* antibodies between July 2010 (and January for HQ) and December 2024. Figure 1a shows the percentages of donors by question response combinations. At CBS, 71% of donors with risk factors were born in Mexico, Central or South America. For about a quarter, their mother or maternal grandmother was born in Mexico, Central or South America, but the donor was not and had not spent 6 months or more there either. At HQ, where different questions were asked, proportionally more of the donors tested were for travel, which was for a shorter duration (1 month HQ vs. 6 months CBS). Figure 1b (CBS only) shows the breakdown of the region of those born in a risk area. Most (71%) were born in South America, and the least (about 11%) were born in Central America, with about 17% in Mexico.

The demographic breakdown of first‐time donors tested for *T. cruzi* antibodies and all other first‐time donors is shown in Tables 1a and 1b with adjusted odds ratios. Donors tested were only slightly different from donors not tested, except they were more likely to be aged 30–49 and to live in a more ethnocultural neighbourhood than non‐tested donors. The odds of first‐time donors being tested have increased over the 15‐year period.

**TABLE 1a:** Demographics of *Trypanosoma cruzi* antibody tested first‐time allogeneic donors versus other first‐time donors (2010–2024), Canadian Blood Services.

	Chagas tested (*N* = 42,546)	All other first‐time donors (*N* = 1,223,129)	Adjusted OR	95% CI
	%	%		
Sex				
Female	52.2	52.7	1.00	
Male	47.8	47.3	0.96	(0.94–0.98)
Age group				
17–29	46.0	48.5	1.00	
30–39	26.4	18.8	1.40	(1.37–1.44)
40–49	16.6	14.7	1.17	(1.14–1.21)
50+	11.0	18.1	0.65	(0.63–0.67)
Region				
British Columbia	18.0	17.0	1.00	
Alberta	17.4	18.3	0.94	(0.91–0.97)
Prairies	8.7	9.6	0.98	(0.94–1.02)
Atlantic	2.8	8.8	0.36	(0.34–0.39)
Ontario	53.2	46.3	1.13	(1.104–1.17)
Material deprivation index^a^				
1 (least deprived)	27.9	26.7	1.00	
2	19.4	22.7	0.82	(0.80–0.85)
3	18.1	20.1	0.82	(0.79–0.84)
4	17.5	17.4	0.84	(0.81–0.86)
5 (most deprived)	17.1	13.2	0.97	(0.93–1.00)
Ethnocultural index^a^				
1 (least ethnocultural)	10.5	16.8	1.00	
2	12.7	19.6	1.02	(0.98–1.06)
3	18.1	21.1	1.27	(1.22–1.32)
4	26.5	21.9	1.70	(1.64–1.77)
5 (most ethnocultural)	32.2	20.6	2.09	(2.01–2.18)
Year				
2010–2014	27.2	33.4	1.00	
2015–2019	36.9	37.0	1.16	(1.13–1.19)
2020–2024	35.9	29.6	1.31	(1.28–1.34)

Abbreviations: CI, confidence interval; OR, odds ratio.

^a^ Indices based on the neighbourhood in which the donor resides.

**TABLE 1b:** Demographics of *Trypanosoma cruzi* antibody tested first‐time allogeneic donors versus other first‐time donors (2010–2024), Héma‐Québec.

	Chagas tested	All other first‐time donors		
	(*N* = 21,914)	(*N* = 398,494)		
	%	%	Adjusted OR	95% CI
Sex				
Female	56.6	56.0	1.00	
Male	43.4	44.0	0.92	(0.90–0.95)
Age group				
17–29	49.2	55.8	1.00	
30–39	23.8	16.8	1.57	(1.51–1.63)
40–49	16.0	13.6	1.33	(1.28–1.39)
50+	11.0	13.9	0.92	(0.88–0.97)
Material deprivation index^a^				
1 (least deprived)	21.6	21.0	1.00	
2	21.2	21.1	0.98	(0.94–1.02)
3	20.1	20.1	0.97	(0.93–1.02)
4	19.0	19.7	0.93	(0.89–0.98)
5	18.2	18.2	0.97	(0.92–1.01)
Ethnocultural index^a^				
1 (least ethnocultural)	10.8	22.5	1.00	
2	11.2	16.8	1.37	(1.29–1.46)
3	23.0	27.3	1.72	(1.64–1.81)
4	26.3	16.4	3.35	(3.18–3.52)
5 (most ethnocultural)	28.6	17.0	3.46	(3.29–3.64)
Year				
2010–2014	28.3	36.2	1.00	
2015–2019	35.1	35.8	1.25	(1.20–1.29)
2020–2024	36.7	28.0	1.58	(1.52–1.64)

Abbreviations: CI, confidence interval; OR, odds ratio.

^a^ Indices based on the neighbourhood in which the donor resides.

The demographic characteristics of first‐time donors who tested positive for *T. cruzi* antibodies are shown in Tables 2a and 2b. There were 27 (21 CBS, 6 HQ) *T. cruzi* positive first‐time donors. Among CBS donors, about half occurred in the first 5 years of testing. About half were from Ontario and none in the Atlantic region; about half were female and about half were 50 years of age or older. In addition, about half were from more materially deprived neighbourhoods (MDI 4 or 5), but only 2 of 21 from the least materially deprived neighbourhoods (MDI 1) and about two‐thirds from more ethnocultural neighbourhoods (ethnocultural index 4 or 5). All but one *T. cruzi* antibody positive first‐time donors had been born in Mexico, Central or South America. The one exception was a 17‐year‐old female donor whose only risk was a mother/grandmother born in a risk area. Among the six HQ donors, most were from ethnocultural neighbourhoods, only one from materially deprived neighbourhoods and none from the least materially deprived neighbourhoods.

**TABLE 2a:** First‐time allogeneic donors who tested positive for *Trypanosoma cruzi* antibodies 2010–2024 with demographic characteristics, Canadian Blood Services.

Donor	Year	Gender	Age	Region	Material deprivation index	Ethnocultural index	Screening risk factors
1	2011	F	19	Ontario	5	5	Birth, mother/grandmother
2	2011	F	55	Prairies	3	4	Birth, travel
3	2011	F	48	Ontario	3	5	Birth, mother/grandmother, travel
4	2012	M	46	Ontario	5	5	Birth, mother/grandmother, travel
5	2012	M	46	Ontario	1	3	Birth, mother/grandmother, travel
6	2013	M	50	Alberta	3	5	Birth, mother/grandmother, travel
7	2014	M	44	Ontario	3	5	Birth, mother/grandmother, travel
8	2014	M	54	Prairies	2	1	Birth, travel
9	2014	M	59	Ontario	4	5	Birth, mother/grandmother, travel
10	2014	F	45	British Columbia	5	5	Birth, mother/grandmother, travel
11	2015	F	39	Prairies	3	4	Birth, mother/grandmother, travel
12	2016	F	50	Prairies	3	2	Birth
13	2018	F	47	Prairies	4	3	Birth, mother/grandmother
14	2018	M	49	Ontario	2	5	Birth, mother/grandmother, travel
15	2018	F	72	Ontario	2	5	Birth, mother/grandmother, travel
16	2019	F	17	Ontario	5	4	mother/grandmother
17	2021	M	71	British Columbia	5	1	Birth
18	2021	M	56	Ontario	5	5	Birth, mother/grandmother
19	2022	F	55	Prairies	1	1	Birth, mother/grandmother, travel
20	2023	F	38	Ontario	4	4	Birth, mother/grandmother, travel
21	2023	M	58	Prairies	4	2	Birth, mother/grandmother

*Note*: Birth = Yes to ‘Were you born in Mexico, Central America of South America?’. Mother/grandmother = Yes to ‘Was your mother or grandmother born in Mexico, Central America of South America?’. Travel = Yes to ‘Have you spent a total of 6 months or more in a continuous period in Mexico, Central America or South America?’.

Abbreviations: F, female; M, male.

**TABLE 2b:** First‐time allogeneic donors who tested positive for *Trypanosoma cruzi* antibodies 2010 to 2024 with demographic characteristics, Héma‐Québec.

Donor	Year	Gender	Age	Region	Material deprivation index	Ethnocultural composition quintiles	Screening risk factor^a^
1	2011	F	38	Quebec	2	4	Unknown
2	2011	M	48	Quebec	2	5	Unknown
3	2016	F	55	Quebec	5	5	Birth, travel
4	2019	F	22	Quebec	3	4	Birth, travel
5	2019	F	36	Quebec	3	1	Birth (travel unknown)
6	2020	M	44	Quebec	3	5	Birth, travel

*Note*: Birth = Yes to ‘Were you or was your mother or maternal grandmother born in Latin America, including Mexico?’ when clarified that the country was in Mexico, Central or South America. Travel = Yes to ‘Have you ever spent time in Latin America (including Mexico) for 30 consecutive days or more?’ when clarified that the country was in Mexico, Central or South America.

Abbreviations: F, female; M, male.

^a^ Data unavailable before 2015.

## DISCUSSION

We have analysed demographic characteristics of all first‐time donors who were tested for *T. cruzi* since selective testing was implemented 15 years ago. Among CBS donors (data not available for HQ) all were born in Latin America except one whose mother or maternal grandmother was born there. Compared with donors who did not require testing, tested donors were more likely to be aged 30–49 and to live in a more ethnocultural neighbourhood. *T. cruzi* positive donors were rare.

Because Chagas disease is primarily a vector‐borne infection, exposure is primarily in Mexico, Central and South America where infection has been endemic since pre‐historic times. The greatest risk of infection is in materially deprived rural areas in individuals living in substandard housing with long‐term triatomine infestation. Insect spraying programmes in Latin American countries targeting such housing have reduced the incidence of infection. At the same time, an exodus of people from rural areas to urban areas within Latin American countries over recent decades has increased the prevalence of Chagas disease in urban areas [1]. That being said, prevalence is not uniform across Latin America, and it is unclear what the risk status of the segment of the Latin American population who immigrate to Canada is [1]. The Latin American community has almost doubled in Canada since 2006, and there are efforts to actively recruit donors of diverse backgrounds, such that some degree of risk from undiagnosed infection is likely to remain [18].

All but one of the first‐time donors who tested positive for *T. cruzi* antibodies were born in Mexico, Central or South America. This is consistent with our previous reports that focused on first‐time tested donors early post‐implementation, and also consistent with reports from other non‐endemic countries where selective testing was implemented [11, 12, 19, 20]. Estimated congenital transmission may occur at rates of about 5% in endemic countries versus 3% in non‐endemic countries, and such infections are often asymptomatic, but regional variation in transmission risk remains poorly understood. Congenital risk may be higher among more recently infected mothers when parasitaemia tends to be higher, and mothers in non‐endemic countries are more likely to have longer time since infection [1]. Thus, people born in Canada with Latin American mothers have a small chance of having undiagnosed Chagas disease. Here we report such a case, a 17‐year‐old donor who had never been to Latin America, and we have previously reported two other cases in our initial post‐implementation report [12].

The duration of travel asked about varies between the two blood operators. At HQ, donors are tested if they have spent 1 month in an endemic country whereas at CBS, it is 6 months; thus, fewer donors are captured by the question. In any event, we did not identify any donors with infection who had travel as their only risk factor, which is consistent with most non‐endemic countries [21].

Most blood donations in Mexico, Central and South America are tested for *T. cruzi*, but in non‐endemic countries selective testing based on screening questions is a cost‐effective option to reduce risk [1, 22]. Selective testing has been implemented in some countries (France, Japan, Luxembourg, New Zealand, Portugal, Spain, Switzerland, Scotland, Canada); others use screening questions without testing, and some have no risk factor criteria [6, 23, 24]. Decisions regarding the strategy depend on the epidemiology of imported Chagas disease in that country. Countries that have implemented selective testing have generally found that infections are very rare in donors, and generally from donors who were born in a risk country. For example, in France, only three donors positive for *T. cruzi* antibodies were identified in the first 18 months of selective testing, all born in South America [20]. In England, only three donors positive for *T. cruzi* antibodies were identified in the first 8 years of selective testing, all born in South America [19]. In Japan, over 6 years of selective testing, three donors were positive, all born or raised in Latin America [25].

In the United States, locally acquired *T. cruzi* infections in humans have been reported in the southern states of Texas, Arizona, California, Louisiana, Mississippi and Missouri [26]. The sylvatic cycle of animals/insect transmission was documented about 150 years ago, but infections in humans in the United States are rare [27]. It is likely that the risk of autochthonous infection will remain confined to the southern United States and not move northwards towards Canada for the foreseeable future due to the complex ecology of triatomines and *T. cruzi* [28]. Due to uncertainty about the level of risk of autochthonous cases in the United States, blood donor screening was initially universal. Infections in blood donors were mostly in those who had been born in Mexico, Central or South America, although a few were likely autochthonous cases born in the United States [8, 29]. The testing strategy in the United States is now one‐time testing of donors to capture the risk of autochthonous cases as well as birth and mother/grandmother birth in Latin America. Indeed, there were very few reports of autochthonous infections prior to the implementation of blood donor testing in the United States [27]. As a high proportion of Chagas disease cases in the United States are believed to be undiagnosed, blood donor screening has proven to make an important contribution to public health surveillance [26, 30].

Our results over nearly 15 years of testing suggest that in Canada, there would be an ongoing chance of *T. cruzi* positive units being released into inventory in the absence of selective testing, albeit small. Country of birth, and on occasion country of birth of mother/maternal grandmother, captured *T. cruzi* antibody positive donations. The criteria for testing based on travel varied between blood establishments (30 days ever vs. 6 months ever). However, as no positive donations were identified among tested donors whose only risk was time spent in risk areas, the value of either blood establishment continuing to test on the respective bases is dubious [21]. Use of selective testing rather than universal testing or selecting all first‐time donors carries a small risk of missing some at‐risk donors. In a study of donors who denied all risk factors, we identified a few who answered screening questions incorrectly (0.8%) [12]. We also identified one donor who had answered questions correctly but had been infected at birth from her mother whose only risk factor was receiving a blood transfusion in Canada, thus missed by current screening questions [5]. However, the rarity of documented transfusion transmission cases worldwide suggests that risk is quite low even without testing. There are several reasons for this. Firstly, in non‐endemic countries donors will be in the indeterminate phase of infection when parasitaemia is low or indetectable. Secondly, viability is reduced with storage time as well as by leucoreduction [31, 32]. Hence, in Canada, both the storage time of red blood cell products and leucoreduction further reduce any risk. Importantly, all confirmed transfusion transmission cases have involved platelet products. Universal pathogen reduction technology is now applied to platelet products at CBS, and HQ is expected to do so in the future [4]. Thus, risk mitigation steps applied to blood products further reduce the low risk of selective testing missing a *T. cruzi* antibody positive donation.

There are some important limitations to our analysis. We are reporting operational data over nearly 15 years of selective testing but without opportunity to collect additional data. There is variability between question algorithms and testing platforms between the two blood operators, but results are consistent across organizations. As it is a selective testing strategy, we do not have test data on donations not captured by risk questions. Donors who test positive for infection are notified, but we do not conduct follow‐up post notification.

In summary, we confirm that the most common risk factor that prompts *T. cruzi* antibody testing is birth in Mexico, Central or South America which captured nearly all positive first‐time donors. Mother or maternal grandmother born in Latin America occasionally yields a positive donation, but travel history does not. Infections in donors are quite rare, but as they continue to be identified over nearly 15 years of monitoring, selective testing interdicts a small risk of *T. cruzi* positive blood products being released into inventory. Therefore, the testing of donors who recently travelled to an at‐risk country provides little to no additional safety benefit. The other testing criteria capture the vast majority of infected donors, although even this benefit may be trivial for blood services that subject platelets to pathogen reduction technology.

## CONFLICT OF INTEREST STATEMENT

The authors declare no conflicts of interest.

## Data Availability

Summary data are available by contacting Dr. Sheila O'Brien (sheils.obrien@blood.ca).
